# Zinc Oxide Nanoparticle Loaded L-Carnosine Biofunctionalized Polyacrylonitrile Nanofibrous Wound Dressing for Post-Surgical Treatment of Melanoma

**DOI:** 10.3390/polym17020173

**Published:** 2025-01-12

**Authors:** Shahin Homaeigohar, Danial Kordbacheh, Sourav Banerjee, Jiacheng Gu, Yilong Zhang, Zhihong Huang

**Affiliations:** 1 School of Science and Engineering, University of Dundee, Dundee DD1 4HN, UK; 2 Division of Cancer Research, School of Medicine, University of Dundee, Dundee DD1 9SY, UK

**Keywords:** wound dressing, polyacrylonitrile nanofibers, zinc oxide nanoparticles, L-carnosine dipeptide, melanoma

## Abstract

Nanofibrous dressing materials with an antitumor function can potentially inhibit recurrence of melanoma following the surgical excision of skin tumors. In this study, hydrolyzed polyacrylonitrile (hPAN) nanofibers biofunctionalized with L-carnosine (CAR) and loaded with bio (CAR)-synthesized zinc oxide (ZnO) nanoparticles, ZnO/CAR-hPAN (hereafter called ZCPAN), were employed to develop an antimelanoma wound dressing. Inspired by the formulation of the commercial wound healing Zn-CAR complex, i.e., polaprezinc (PLZ), for the first time, we benefitted from the synergy of zinc and CAR to create an antimelanoma nanofibrous wound dressing. According to scanning electron microscopy (SEM) images, ultrafine ZnO nanoparticles were homogenously distributed throughout the nanofibrous dressing. The ZCPAN nanofiber mat showed a significantly higher toughness (18.7 MJ.m^−3^ vs. 1.4 MJ.m^−3^) and an enhanced elongation at break (stretchability) compared to the neat PAN nanofiber mat (12% vs. 9.5%). Additionally, optical coherence elastography (OCE) measurements indicated that the ZCPAN nanofibrous dressing was as stiff as 50.57 ± 8.17 kPa which is notably larger than that of the PAN nanofibrous dressing, i.e., 24.49 ± 6.83 kPa. The optimum mechanical performance of the ZCPAN nanofibers originates from physicochemical interaction of CAR ligands, hPAN nanofibers, and ZnO nanoparticles through hydrogen bonding, electrostatic bonding, and esterification, as verified using ATR-FTIR. An in vitro cell viability assay using human skin melanoma cells implied that the cells are notably killed in the presence of the ZCPAN nanofibers compared to the PAN nanofibers. Thanks to ROS generating ZnO nanoparticles, this behavior originates from the high reactive oxygen species (ROS)-induced oxidative damage of melanoma cells, as verified through a CellROX assay. In this regard, an apoptotic cell response to the ZCPAN nanofibers was recorded through an apoptosis assay. Taken together, the ZCPAN nanofibers induce an antimelanoma effect through oxidative stress and thus are a high potential wound dressing material to suppress melanoma regrowth after surgical excision of skin tumors.

## 1. Introduction

Melanoma, as a highly aggressive skin disease, causes 75% of the deaths related to skin cancer [1]. Melanoma is traditionally treated through surgical excision of skin tumor [2]. Nevertheless, the surgery is usually followed by tumor relapse, negatively affecting the therapeutic outcome and raising the mortality chance. Additionally, the surgery leads to a skin wound, which challenges prognosis after the removal of the tumor [3]. To inhibit the regrowth of residual tumor after surgery, radiotherapy and chemotherapy are extensively applied [4,5]. However, they induce painful side effects and resistance to therapy in patients. For instance, chemotherapy deals with systemic administration of drugs to stop tumor growth. This treatment imposes detrimental consequences to different organs, thereby reducing the antitumor efficiency of the drug [3]. As a new strategy, advanced wound dressings have been designed to offer an antitumor function and accelerate the wound healing process. In this regard, it is crucial to develop wound healing materials that can not only promote wounded skin regeneration, but also inhibit bacterial infection and metastasis and recurrence of the melanoma after surgical resection. For instance, Wang et al. [6] incorporated copper sulfide (Cu_2_S) nanoflowers into a micro-patterned nanofibrous material composed of polylactic acid (PLA)/polycaprolactone (PCL) blend. The Cu_2_S nanoflowers induced a photothermal effect when irradiated with near infrared (NIR) light, thereby killing skin tumor cells efficiently (>90%). As another example, Yuan et al. [3] developed a nanofibrous wound dressing based on 5-fluorouracil loaded mesoporous bioactive glass particle reinforced poly(lactic-co-glycolic acid) (PLGA) nanofibers for the post-surgical treatment of melanoma. This nanofibrous dressing steadily released the incorporated chemotherapeutic drug into the wound milieu. As a result of the nanostructure, formulation, and drug delivery of the nanofibrous wound dressing, not only was tumor growth suppressed, but also skin regeneration was supported.

Multifunctional nanofibrous wound dressings are demanded for the treatment of surgical wounds after tumor resection or excision as they deal with several complications such as leftover cancer cells, infection, and poor healing capacity. In our recent study [7], we validated the wound healing efficiency and antibacterial activity of a new nanofibrous wound dressing material comprising ZnO nanoparticles, L-carnosine dipeptide, and hydrolyzed PAN nanofibers. This biohybrid nanomaterial stimulates the proliferation, adhesion, and migration of endothelial cells and fibroblast cells that play a decisive role in the wound healing process. Additionally, the ZnO/CAR-hPAN (ZCPAN) nanofibers were shown to effectively inhibit the activity of *Staphylococcus aureus* bacteria. In this study, the antimelanoma potential of the ZCPAN nanofibrous wound dressing was investigated. To the best of our knowledge, a biohybrid antitumor nanofibrous dressing in which the surface loaded biological agent can chelate metal ions, thereby forming anticancer metal oxide nanoparticles is totally novel.

Advantageous over natural polymers such as alginate, chitosan, collagen, among others, synthetic polymers (e.g., PAN) offer promising physicochemical properties (e.g., tunable mechanical stiffness and surface chemistry); they are typically low cost and scalable, and can easily be processed and integrated into industrial technologies [8]. These engineering characteristics play a significant role in the translation of synthetic polymer made wound dressings to clinical products. Moreover, as a synthetic non-degradable polymer, PAN does not induce fibrotic and inflammatory responses in skin. This contrasts with synthetic biodegradable polymers (e.g., PLGA and PLA) that are commonly employed for the development of wound dressings [9]. Additionally, electrospinning of PAN is straightforward compared to that of natural polymers that typically need hazardous catalysts and/or chemical cross-linkers. Therefore, PAN can be readily nanofabricated through advanced electrospinning technology at large scale. Despite the superior physicochemical properties of PAN nanofibers, they poorly interact with cells. To overcome this hurdle, we surface treated the hydrolyzed PAN nanofibers with CAR, an endogenous dipeptide (comprising β-alanine and l-histidine) [7]. CAR is produced in the cells with long lifespan, e.g., nerve cells and muscle cells [10], and acts as an antioxidant, as well as an antiglycan and antiaging reagent [11]. CAR can play a crucial role in the healing of surgical wounds [12], e.g., those remaining after the removal of skin tumors, due to its contribution to the generation of nitric oxide (NO) by endothelial cells [13] and to the longevity of fibroblasts [14]. According to McFarland and Holliday [15], CAR promotes the viability and growth of human fibroblast cells (strains HFF-1 and MRC-5). In a CAR rich cell medium, the phenotype of fibroblast cells changes to juvenile, while it is senescent in a CAR free medium. This phenotypic variation is reflected in further colonization and growth of the cells, as was observed in our study [7]. Hipkiss et al. [16] attributed this supportive role of CAR to its extensive reaction with sugars, thereby hampering non-enzymic protein glycosylation. In addition to the promising wound healing potential of CAR, it can chelate metal ions. Nature-derived chelating agents have been proven effective for a variety of advanced applications such as sensing and disinfection [17]. The metal-chelating activity of CAR could be employed for the biosynthesis of antitumor metal oxide agents. In our recent study [7], we proved experimentally that ZnO nanoparticles are biosynthesized on the surface of the CAR biofunctionalized PAN nanofibers. ZnO nanoparticles have been shown to produce reactive oxygen species (ROS), thereby inducing apoptosis in cancer cells [18]. Therefore, in the current study, ZnO loaded CAR biofunctionalized hPAN (ZCPAN) nanofibers were employed to develop an antimelanoma wound dressing. While the wound healing and antibacterial capacity of the ZCPAN nanofibers were validated in our earlier study [7], their antimelanoma effect was thoroughly investigated here.

## 2. Materials and Methods

### 2.1. Materials

PAN (Mw = 150,000; CAS No. 25014-41-9), L-carnosine (99%, crystalline, Mw = 226.23 CAS No. 305-84-0), zinc chloride (ZnCl_2_) (reagent grade, ≥98%, CAS No. 7646-85-7), sodium hydroxide (NaOH) (ACS reagent, ≥97.0%, pellets, CAS No. 1310-73-2), and N, N-dimethylformamide (DMF) (anhydrous, 99.8%, CAS No. 68-12-2) were purchased from Sigma-Aldrich (Darmstadt, Germany). Phosphate buffered saline (PBS) was obtained from Carl Roth GmbH (Karlsruhe, Germany). All chemicals and materials were used as received.

### 2.2. Preparation and Functionalization of PAN Nanofibers

PAN nanofiber mats were prepared via electrospinning of a PAN/DMF solution (8 wt% (*w*/*w*)) using an EF-100 electrospinning machine (SKE Research Equipment, Italy). The solution was fed into a syringe needle by a syringe pump at the feed rate of 0.8 mL/h. The PAN nanofibers were deposited on an aluminum foil 25 cm distant from the needle under a voltage of 15 kV at room temperature. The collected nanofibers were vacuum dried overnight at 100 °C to evaporate the residual solvent.

The PAN nanofibers were chemically treated (hydrolyzed) by immersion in an NaOH solution (1 N) for 2 h at 60 °C. The hydrolyzed PAN (hPAN) nanofibers were subsequently rinsed with distilled water and air dried. The hPAN nanofibers were immersed into a CAR/PBS solution (5 mg/mL) and shaken for 5 h at 50 °C. The CAR-hPAN nanofibers were washed with PBS and air dried. The CAR-hPAN nanofibers were eventually loaded with ZnO nanoparticles by soaking them in a ZnCl_2_ aqueous solution (20 wt%) for 2 h at ambient temperature. The ZnO/CAR-hPAN nanofibers were rinsed thrice using deionized water and air dried.

### 2.3. Morphological and Physicochemical Characterization

The surface morphology of neat PAN, CAR-hPAN, and ZnO/CAR-hPAN nanofibers was observed with scanning electron microscopy (SEM) (Zeiss Sigma VP Gemini from Carl ZEISS, Jena, Germany) under a 10 kV electron accelerating voltage. The nanofibers were previously sputter coated with Au/Pd nanoparticles.

Surface chemistry of neat PAN, CAR-hPAN, and ZnO/CAR-hPAN nanofibers was characterized through ATR-FTIR spectroscopy (ALPHA (ATR-Ge, ATR-Di) from BRUKER Optik GmbH, Ettlingen, Germany). For this purpose, over a spectral range of 400–4000 cm^−1^, 40 spectral scans (with a resolution of 4 cm^−1^) were averaged.

The tensile toughness and elongation at break (stretchability) of the neat PAN (control) and ZnO/CAR-hPAN nanofiber mats (3 cm × 1 cm × 0.1 cm) were determined by using a uniaxial tensile tester (Universal Tester Instron 4204, Canton/ MA, USA, 1000 N load cell). The measurements were repeated five times (five nanofiber mats).

### 2.4. Optical Coherence Elastography (OCE)

A custom-built vibrational OCE equipment, consisting of a phase-sensitive optical coherence tomography (PhS-OCT) system and an external vibration excitation system, was utilized to characterize the elasticity distribution within the PAN (control) and ZCPAN nanofiber mats. The description of the applied system and elasticity estimation method are detailed in [19]. Briefly, the main components of the PhS-OCT system included a broadband laser source (Thorlabs Inc., Newton, NJ, USA) with a center wavelength of 1310 ± 110 nm and a high-speed InGaAs linear array camera with a maximum sampling frequency of 91,912 Hz (SUI, Goodrich Corp, Charlotte, NC, USA) for capturing the interference signal spectrum. The external vibrational excitation system comprised a mechanical shaker (Brüel & Kjær Sound & Vibration Measurement A/S, Copenhagen, Denmark) driven by an externally triggered function generator (Keysight Technologies Inc., Santa Rosa, CA, USA), which applies periodic vibration to the sample oriented along the vertical axis at the shaker’s head. The axial and lateral resolutions of the vibrational OCE system were calculated to be approximately 6.9 μm and 12.4 μm in air, respectively. The imaging depth was measured to be 2.8 mm in air, and the system demonstrated a displacement sensitivity of 2 nm.

In this study, an agar-based tissue mimicking solution (1% *w*/*v*) was dropwise deposited onto the nanofiber surface and then air dried to form a thin layer (with an elastic modulus of 14.60 ± 0.80 kPa, measured via a conventional compression test). In the next step, the excitation system applied a sinusoidal signal with a frequency of 750 Hz and an amplitude of 800 mVpp to the nanofibers. A M-B scanning protocol was employed to detect the nanofibers’ response to the periodic vibration, with the A-line rate of 10,360 Hz and frame rate of 20 Hz, thereby enabling motion detection in the vibrational OCE. Each acquisition took approximately 25 s. To ensure repeatability of the results, the measurements were carried out on three PAN and ZCPAN nanofibrous mats. In these measurements, the effective imaging depth was ~2 mm, and the effective lateral distance was set at 12 mm with a sampling distance of 23 μm.

### 2.5. Melanoma Cell Culture

A375-M2 malignant melanoma cells were purchased from ATCC (CRL-3223) and cultured in Dulbecco’s Modified Eagle’s Medium supplemented with 10% fetal bovine serum together with penicillin and streptomycin. The cells were maintained at 5% CO_2_ and 37 °C in a cell culture incubator. Cells numbering 100,000 were loaded into a single well of a 24-well plate and were allowed to acclimatize for 24 h. Following this, the cells were treated with the PAN and ZCPAN nanofibers. The nanofibers were previously sterilized under UV light for 1 h.

For SEM imaging of cell morphology on the nanofiber mats, the cells were seeded on the ZCPAN nanofibers, incubated for 24 h, and eventually were fixed in 10% formalin overnight. The ZCPAN nanofibers were air dried, and sputter coated with Au nanoparticles. The cell morphology was imaged using SEM (JEOL JSM, 7400F, Tokyo, Japan) under an electron accelerating voltage of 5 kV.

### 2.6. Melanoma Cell Viability

The A375-M2 cells treated with the PAN and ZCPAN nanofibers for 24 h were analyzed in terms of cell viability through a previously explained assay using CellTiter 96^®^ AQueous Non-Radioactive Cell Proliferation Assay kit (Promega, Madison, WI, USA) [20,21]. The optical absorbance was measured in a Tecan multi-well plate reader and the obtained data represented as relative to a no-treatment control. To further analyze if the exposure of the cells to the ZCPAN nanofibers induces apoptotic cell death, Annexin V-FITC (ab176749; Abcam, Cambridge, UK) was added to the wells following the manufacturer’s instructions. FITC fluorescence was captured in a Tecan multi-well plate reader and the obtained data represented as relative to no-treatment control.

### 2.7. ROS Quantification Assay

The ROS production of the ZCPAN nanofibers and their detrimental effect on the A375-M2 cells were monitored using CellROX Green (#C10444; ThermoFisher Scientific, Waltham, MA, USA) [22] and following the manufacturer’s instructions.

### 2.8. Statistical Analysis

The statistical analysis of biological data was carried out through *t*-test and using one-way analysis of variance (ANOVA) while using Graphpad Prism 9 statistical package. The *p*-values lower than 0.05 represented a significant difference in the biological data series.

## 3. Results and Discussion

**Figure 1:**
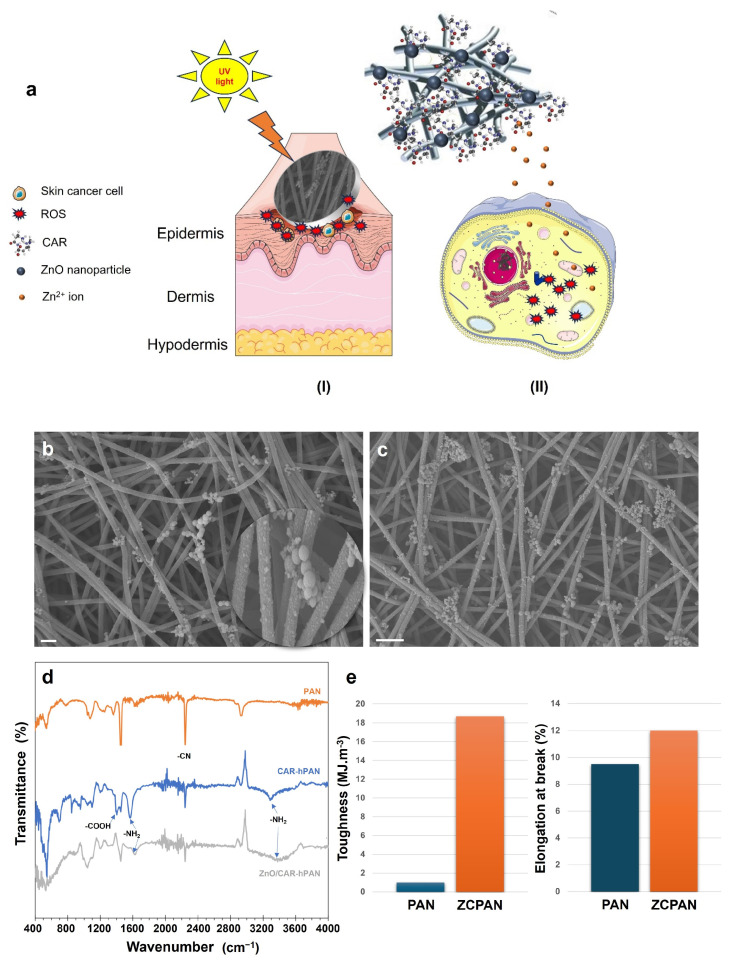
(**a**) Schematic illustration of the mechanisms of anticancer action of the ZCPAN nanofibrous wound dressing: (I) UV-light irradiation drives ROS generation by the ZnO nanoparticles located on the CAR-hPAN nanofibers (excessive ROS density is lethal to skin cancer cells), or (II) Dissolution of the ZnO nanoparticles on the ZCPAN nanofibers, and thus the release of Zn^2+^ ions, stimulates mitochondria to generate intracellular ROS, thereby inducing mitochondrial dysfunction and cell apoptosis (graphical items of skin and cell were obtained from Servier Medical Art, CC-BY4.0). SEM images show the uniform distribution of ZnO nanoparticles throughout the ZCPAN nanofiber mats (scale bars represent 1 µm (**b**) and 2 µm (**c**)). The inset image (**b**) highlights the synthesis of ultrafine, homogenously distributed ZnO nanoparticles along the CAR-hPAN nanofibers. (**d**) ATR-FTIR spectra of neat PAN, CAR-hPAN, and ZnO/CAR-hPAN nanofibers. The obvious shift or disappearance of the characteristic bands of the CAR-hPAN nanofibers implies their significant Zn^2+^ ion chelating activity (coordinate bonding). (**e**) Toughness and elongation at break (stretchability) of neat PAN and ZCPAN nanofiber mats.

Thanks to the presence of ZnO nanoparticles and their ROS releasing activity, it was assumed that the ZCPAN (ZnO/CAR-hPAN) nanofibers would be able to kill melanoma cells, thus acting as an antimelanoma wound dressing material. Figure 1a schematically illustrates the mechanisms of the anticancer function of the ZCPAN nanofibrous wound dressing. Under UV-light irradiation, ZnO nanoparticles generate ROS that potentially kill tumor cells thus allowing for the post-surgical treatment of melanoma. Moreover, the likely dissolution of the ZnO nanoparticles in the acidic medium of the tumor leads to the release of Zn^2+^ ions that stimulate mitochondria to generate intracellular ROS which cause cell apoptosis. Uniform release of ROS/Zn^2+^ within the wound necessitates homogenous distribution of ZnO nanoparticles throughout the nanofibrous dressing. As shown in Figure 1b,c, due to the remarkable metal ion chelating activity of CAR, ultrafine ZnO nanoparticles are biosynthesized across the nanofibrous mat in a uniform manner. Despite the formation of some ZnO clusters, the majority of the ZnO particles are nanoscale and non-agglomerated (as evidently shown in the inset image). This promising observation stems from uniform biofunctionalization of the hydrolyzed PAN nanofibers that maintain the high biofunctionality of CAR and its metal ion chelating capacity.

The proper biofunctionalization of the hPAN nanofibers can be validated through ATR-FTIR spectroscopy (Figure 1d). According to our earlier studies [7,8], hydrolysis of PAN nanofibers leads to the formation of carbonyl and hydroxyl functional groups on the surface of the nanofibers which can physiochemically bond with CAR’s functional groups (carboxyl and amine). In the pristine form, the characteristic bands of CAR appear at 3240 cm^−1^ (amine), 1585 cm^−1^ (amine), and 1404 cm^−1^ (carboxyl) [23]. While, for the CAR-hPAN nanofibers, these bands shift to 3290 cm^–1^ (amine), 1570 cm^–1^ (amine), and 1400 cm^–1^ (carboxyl), implying their hydrogen bonding with the functional groups of the h-PAN nanofibers. On the other hand, while the hydroxyl groups of the h-PAN nanofibers are represented by a characteristic band at 1355 cm^–1^ [8], this band disappears upon CAR immobilization, most likely due to esterification of OH groups (hPAN) and carboxyl groups (CAR). Synergistically, hydrogen bonding and esterification of CAR and h-PAN leads to stable, uniform immobilization of CAR on the hPAN nanofibers. As seen in Figure 1d, compared to the PAN nanofiber’s FTIR spectrum, the intensity of the nitrile band (2243 cm^−1^) drastically declines in the CAR-hPAN nanofibers’ spectrum, while new carboxyl (1400 cm^−1^) and amine (3290 cm^−1^ and 1570 cm^−1^) bands emerge. This abundance of electron donating functional groups provides the CAR-hPAN nanofibers with a strong metal ion (Zn^2+^) chelating potential, as reflected in the FTIR spectrum of the ZnO/CAR-hPAN nanofibers. While no specific characteristic bands of ZnO are detectable, CAR’s amine and carboxyl bands almost vanish or shift. Such a major change originates from the coordinate bonding of Zn^2+^ ions and exposed functional groups of the CAR-hPAN nanofibers. Taking the remaining functional groups of the hPAN nanofibers aside, CAR’s functional groups through carboxylate oxygen, deprotonated amide N-atom and/or terminal amino N-atom can chelate Zn^2+^ ions [23]. In a metal–CAR complex, the metal ions bind to two N atoms of CAR’s imidazole groups, and carboxyl O and amino N atoms of CAR through four coordination bonds [24,25]. While Zn nanoparticles are expected to form as a result of metal ion chelation, XRD results (shown in our earlier study [7]) indicated that ZnO nanoparticles coat the surface of the CAR-hPAN nanofibers. The dissolved oxygen in zinc chloride aqueous solution (or even atmospheric oxygen when the nanofibers are dried) could be responsible for the formation of ZnO nanoparticles. Similarly, Wu et al. [26] stated that ZnO nanocrystals can be biosynthesized through incubation of Zn^2+^ with aqueous solutions of human serum, rat serum, and bovine serum.

Mechanical toughness and stretchability of wound dressings are crucial to ensure that the rupture of the dressing material, particularly on the stretchable parts of the body with frequent motion of skin, e.g., joints, does not cause secondary injury and delay healing [27]. As seen in Figure 1e, the ZCPAN nanofibers show a significantly higher toughness (18.7 MJ.m^−3^ vs. 1.4 MJ.m^−3^) and larger elongation at break (stretchability) (12% vs. 9.5%) compared to the neat PAN nanofibers. This promising ductility might originate from the robust attachment of ZnO nanoparticles and CAR on the hPAN nanofibers. Particularly, the interfiber cross-linking role of CAR ligands can largely improve the toughness and stretchability of the ZCPAN nanofibrous dressing. Similarly, improved mechanical stability of a biofunctionalized poly(acrylonitrile-co-glycidyl methacrylate) (PANGMA) nanofibrous membrane mediated by the presence of bovine serum albumin protein nanowires, which act as an interfiber cross-linker, has already been reported in the literature [28]. As discussed earlier in the FTIR section, CAR ligands were stabilized on the hPAN nanofibers through hydrogen bonding (hydroxyl–amine) and esterification (hydroxyl–carboxyl). The large number of CAR ligands throughout the nanofibrous membrane provides a significant bonding between the nanofibers and CAR ligands from adjacent nanofibers, thereby maximizing the mechanical robustness of the ZCPAN nanofibers. Additionally, ZnO nanoparticles are typically hydroxylated (thanks to air humidity or water interface) [29] and could further strengthen the nanofibrous mat through their involvement in esterification and hydrogen bonding with the CAR molecules of the same nanofiber they belong to, or those of adjacent nanofibers. According to Blok and de Bruyn [30], the water contacting ZnO surface can be characterized as Zn(OH)_2_ with an ionic double-layered structure. The presence of an ionic double surface layer is validated by monitoring the pH changes of the solution surrounding ZnO, implying the release or consumption of OH^-^ ions as a result of the trade-off between anions in solution and surface hydroxyls. Therefore, the ZnO nanoparticles also carry a negative electrostatic charge at neutral pH which enables them to interact electrostatically with positively charged amine rich CAR molecules (with the isoelectric point of 8.2 [31]) and further enhance the toughness of the ZCPAN nanofibrous dressing.

**Figure 2:**
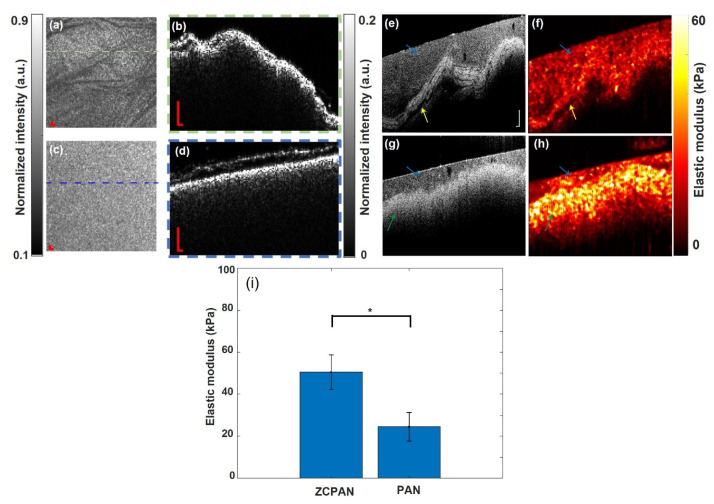
Representative mean intensity projection and cross-sectional OCT structural images of the ZCPAN and PAN nanofiber mats: en face image of the PAN (**a**) and ZCPAN (**c**) nanofiber mats (green and blue dashed lines represent the location of the cross-sectional image) and cross-sectional image of the PAN (**b**) and ZCPAN (**d**) nanofiber mat (scale bar = 100 µm). OCT structural and corresponding vibrational elastography images of the ZCPAN and PAN nanofiber mats: OCT structural image of the PAN (**e**) and ZCPAN (**g**) nanofiber mat and corresponding elastic modulus distribution map of the PAN (**f**) and ZCPAN (**h**) nanofiber mat (blue, green, and yellow arrow indicate agar phantom, ZCPAN and PAN nanofibers, respectively) (scale bar = 200 µm). Evidently, a larger number of highly stiff zones are found across the ZCPAN nanofiber mat compared to the PAN nanofiber mat which plays a supportive role for cell adhesion. (**i**) The elastic modulus of the ZCPAN and PAN nanofibrous mats determined through OCE (*: *p* < 0.05).

The interaction of cells and nanofiber biomaterials largely depends on the biochemistry and biomechanics of the nanofibrous structures. With respect to biomechanics, due to a complex composition and structure, natural tissues such as skin are considered as non-linear viscoelastic solids with anisotropic mechanical properties which are highly dependent on the tissue’s micro/macroscopic structural organization [32]. However, wound dressing materials are preferred to offer isotropic mechanical responses through a homogenous structure to uniformly provoke or inhibit cellular activity. In the current study, optical coherence tomography (OCT), as a non-destructive and ultra-fast imaging technology, was employed to visualize the homogeneity of the surface and cross-section of the ZCPAN and PAN nanofiber mats. Additionally, the elastic modulus of the nanofiber mats was mapped with high resolution using our unique dynamic vibrational elastography (OCE) technique. This stiffness mapping of the nanofibrous mats with micron resolution might give new insight into the intricate cell–nanofiber interactions leading to regeneration of a natural tissue like skin. To the best of our knowledge, this is the first application of OCT and its functional elastography (OCE) in the characterization of nanofibrous biomaterials. The representative OCT structural images of the ZCPAN and PAN nanofiber mats are shown in Figure 2a–d. The en face OCT images of the PAN (Figure 2a) and ZCPAN (Figure 2c) nanofiber mats were generated by the mean intensity projection of a 1.8 mm depth range of the OCT structural volume. The cross-sectional OCT structural images of the PAN (Figure 2b) and ZCPAN (Figure 2d) nanofiber mats were highlighted in the corresponding projection images. The ZCPAN nanofiber mat exhibited a uniform morphology, whereas the PAN nanofiber mat’s projection images featured undulation patterns. The inner-structural information revealed that the surface of the ZCPAN nanofiber mat was flat with homogeneously distributed nanofibers. In contrast, the surface of the PAN nanofiber mat was wrinkled, and more importantly, pores were visible across the mat. Clearly, the ZCPAN nanofiber mat is much more homogenous than the PAN nanofiber mat and thus can provide a relatively more isotropic mechanical response to the cells (melanoma or healthy cells).

Figure 2e–h shows the inner structural and vibrational elastography images of the ZCPAN and PAN nanofiber mats. An agar-based tissue-mimicking phantom (marked with a blue arrow) was used as a reference for estimating the actual elastic modulus of the nanofibers. In the structural images, the ZCPAN nanofiber mat (Figure 2g) exhibited more homogeneous properties compared to the PAN nanofiber mat (Figure 2e). The corresponding elastography images (Figure 2f,h) displayed high-resolution elastic modulus distributions, with brighter colors indicating higher elastic modulus values. The agar layer showed a homogeneous elastic modulus in both elastography images. The ZCPAN nanofiber mat demonstrated an elastic modulus of approximately 50 kPa. In contrast, the elastic modulus of PAN was significantly lower, comparable to that of the agar phantom.

Figure 2i shows the average elastic modulus of the PAN and ZCPAN nanofiber mats, obtained through the elastography images. The elastic modulus of the ZCPAN nanofiber mat was determined to be 50.57 ± 8.17 kPa, which was significantly higher than that of the PAN nanofiber mat, i.e., 24.49 ± 6.83 kPa (*p* < 0.05). Biofunctionalization of the nanofibers and biosynthesis of ZnO nanoparticles on the PAN nanofibers lead to a notably larger (almost 100%) stiffness in the ZCPAN nanofiber mat compared to the PAN nanofiber mat. As discussed earlier, this improved mechanical performance of the ZCPAN nanofiber mat stems from the physicochemical interaction of the components of this nanofiber formulation.

Alongside biochemistry of the nanofibers, the local elasticity of nanofibrous scaffolds plays a major role in cellular activities such as differentiation, proliferation, and migration [33]. Cells apply forces on the scaffold to initiate migration, thereby remodeling its structure over the course of tissue regeneration [34]. During each step of tissue regeneration, cells sense their neighboring mechanical medium through surface mechanoreceptors [33]. Upon touching the scaffold surface, cell integrins attach to the surface ligands of the scaffold and create large protein complexes, known as focal adhesions, which secure the attachment of the cell cytoskeleton to the scaffold. Such a process is decisive in adhesion, morphology, and the function of cells [35]. Focal adhesions continuously sense the stiffness of the underlying scaffold and perform as local nanosensors [36]. To enable the cells to persistently migrate in a specific direction, the scaffold must be stiff enough to withstand cell traction induced deformation. On a 2D planar substrate, e.g., a nanofibrous mat, cells travel toward stiff areas. This process is called durotaxis [37]. Therefore, a stiffer ZCPAN nanofiber mat would be more encouraging for both cancer cells and normal cells to adhere and migrate onto. As a result, cells interact with the underlying nanofibrous substrate to a larger extent, thereby promoting wound healing by normal cells and maintaining the cancer cells in close proximity to the ZnO nanoparticles to assure ROS-induced apoptosis. Additionally, in terms of wound healing, it is known that the molecular pathways responsible for cell proliferation are also modulated through the contractile forces transferred by the actomyosin network, which are eventually sensed by focal adhesions. The level of the contractile forces largely depends on the elasticity of the 2D substrate [38], e.g., a nanofiber mat. Therefore, the ZCPAN nanofiber mat is believed to be more supportive towards cell proliferation and thus wound healing.

As the main objective of the current study, the ZCPAN nanofibers were challenged in terms of antimelanoma activity. In this novel nanoformulation, the ZnO nanoparticles phase is the only one that has been extensively shown in the literature to offer anticancer properties [18,39,40]. ZnO nanoparticles belong to a well-known class of metal oxide nanoparticles that features a variety of promising physicochemical and biological characteristics including strong NIR and UV absorption, antibacterial activity, optimum catalytic potential, and mechanical stability [41]. ZnO is a n-type semi-conductor with a high excitation binding energy of 60 meV and a wide direct energy band gap of 3.37 eV [42]. ZnO nanoparticles can be activated by UV light (and even by visible light due to their intrinsic defects) to generate persistent excitons which subsequently create ROS in the presence of water and oxygen [42], e.g., hydrogen peroxide (H_2_O_2_), hydroxyl radical (^•^OH), singlet oxygen (^1^O_2_), and superoxide anion radical (^•^$O_{2}^{-}$) [43]. These ROS are produced through the redox reactions driven by photoinduced holes (h^+^) and electrons (e^−^) on the surface of ZnO nanoparticles [43].

**Figure 3:**
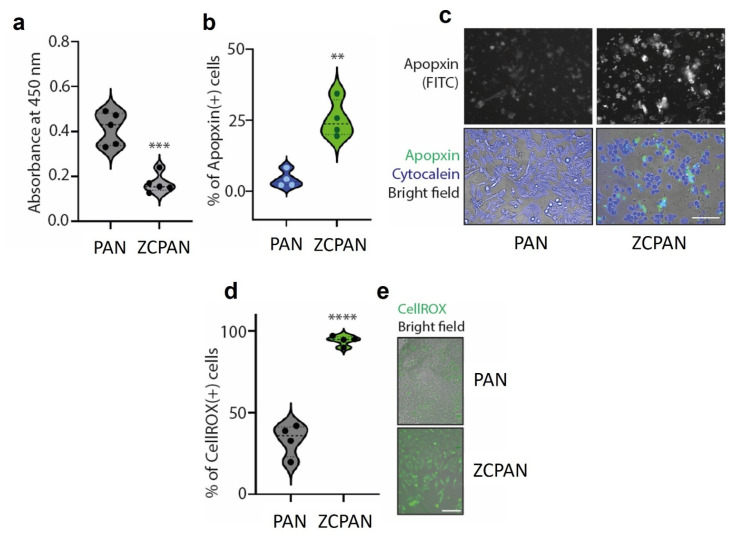
(**a**) Viability of the A375-M2 melanoma cells treated with the ZCPAN (ZnO/CAR-hPAN) nanofibers after 24 h incubation. Compared to the control (PAN) nanofibers, a significantly decreased cell viability is caused by the ZCPAN nanofibers (***: *p* < 0.001). (**b**) The percentage of apopxin positive cells (the cells that underwent apoptosis) in the presence of the ZCPAN nanofibers significantly prevails over that with the control (PAN) nanofibers (**: *p* < 0.01). (**c**) Bright field microscopy images of the live and apoptotic melanoma cells treated with the PAN and ZCPAN nanofibers for 24 h. In these images, the live and apoptotic cells were stained with Cytocalcein Violet 450 (blue spots) and Apopxin Green indicator (green spots), respectively. The apoptotic cells were also visualized using the FITC channel (upper row). Clearly, the number of apoptotic cells (apopxin stained) treated with the ZCPAN nanofibers is much higher than that with the PAN nanofibers. In contrast, a larger density of live cells (Cytocalcein stained) is seen around the PAN nanofibers compared to the ZCPAN nanofibers. The morphology of the apoptotic cells changes to spherical compared to the spindle-like shape of live cells (mainly seen in the control group). (**d**) The CellROX assay implies the significantly increased intracellular ROS levels in the melanoma cells treated with the ZCPAN nanofibers for 8 h compared to those treated with the PAN nanofibers (****: *p* < 0.0001). (**e**) Bright field microscopy images of CellROX green stained melanoma cells in the presence of the PAN and ZCPAN nanofibers. A clearly larger number of ROS containing melanoma cells is found after treatment with the ZCPAN nanofibers. All experiments were carried out in four or five biological replicates.

The ROS generated by ZnO nanoparticles (particularly hydroxyl radicals) play a significant role in DNA damage, apoptosis, and other cellular processes [39]. Therefore, it is postulated that the ZCPAN nanofibers might show an anticancer activity, thanks to the presence of ZnO nanoparticles. This postulate was investigated through a cancer cell viability test. As shown in Figure 3a, the viability of the A375-M2 cells significantly declines in the presence of the ZCPAN nanofibers compared to the PAN nanofibers (control) (*p* < 0.001). The cancer cell death could be mainly ascribed to oxidative stress caused by the ZnO nanoparticles originated ROS that leads to apoptosis.

As seen in Figure 3b, the number of apopxin positive cells, i.e., the cells that underwent apoptosis, is much higher in the presence of the ZCPAN nanofibers compared to the PAN nanofibers (control) (*p* < 0.01). The ROS derived from the ZCPAN nanofibers largely damage the DNA of the melanoma cells, thereby inducing apoptotic pathways by secreting apoptogenic factors from the mitochondrial inter-membrane space [39]. As a result, apoptosomes form that can trigger executioner enzymes [44] leading to apoptosis and cell death. The ROS can be produced not only by the UV-irradiated ZnO nanoparticles (of the ZCPAN nanofibers), but also as a result of dissolution of ZnO in an acidic medium [45]. It is worth noting that due to cell metabolism, the cell culture medium becomes acidic over a specific time [46]. The dissolution product of the ZnO nanoparticles, i.e., Zn^2+^ ions stimulate mitochondria to produce additional ROS which cause mitochondrial dysfunction and eventually apoptosis [40]. The second pathway, i.e., Zn^2+^ induced generation of intracellular ROS, seems to be more realistic in the case of the ZCPAN nanofibers as they were UV irradiated (as part of the sterilization process) before immersion in the A357 cell culture medium. There is also another mechanism for ROS generation via activation of nicotinamide adenine dinucleotide phosphate (NADPH) oxidase enzyme that produces ^•^$O_{2}^{-}$ in the membrane of phagocytic cells [39]. This is plausible only if the ZnO nanoparticles are released into the culture medium which is unlikely due to the strong coordinate bond between CAR and ZnO nanoparticles. Therefore, there are other chemical pathways which enable ROS generation by the ZnO nanoparticles even in the dark. To further verify the pro-apoptotic activity of the ZCPAN nanofibers, the melanoma cells treated with these nanofibers for 16 h were stained and imaged by bright field microscopy. Figure 3c visualizes the apoptotic and live cells stained with Apopxin Green indicator and Cytocalcein Violet 450, respectively. Clearly, the number of apoptotic cells is significantly larger in the presence of the ZCPAN nanofibers compared to that with the PAN nanofibers. In contrast, with the PAN nanofibers, almost all the cells are alive. Similar to our observation, Sharma et al. [47] also reported apoptosis mediated changes in human primary epidermal keratinocytes that are exposed to ZnO nanoparticles.

The anticancer action of the ZCPAN nanofibers is mainly attributed to oxidative damage of the melanoma cells. As seen in Figure 3d, the number of intracellular ROS containing melanoma cells (CellROX positive cells) treated with the ZCPAN nanofibers is significantly larger than that with the PAN nanofibers (*p* < 0.0001). This was also confirmed by the bright field microscopy images (Figure 3e) which visualize the abundance of the melanoma cells with intracellular ROS after exposure to the ZCPAN nanofibers. The ROS produced directly (upon UV irradiation) or indirectly (Zn^2+^ mediated) by ZnO nanoparticles can activate the redox-cycling cascade in the cancer cells or in their membrane, thereby depleting cellular antioxidants and eventually imposing irreparable oxidative damage [39,48]. According to Alarifi et al. [39], in the cells exposed to ZnO nanoparticles, while the amount of ROS and membrane lipid peroxidation notably rises, the amount of the antioxidant molecule, i.e., glutathione, and the antioxidant enzymes, i.e., superoxide dismutase and catalase, drastically declines. After exposure of cancer cells to ZnO nanoparticles, glutathione which is an antioxidant cellular tripeptide is largely used up [39]. Additionally, superoxide dismutase, which transforms extremely toxic superoxide radicals to less toxic hydrogen peroxide, and the catalase enzyme, which reduces hydrogen peroxide to water, are depleted [39]. Therefore, a combination of several factors including increased generation of intracellular ROS, enhanced membrane lipid peroxidation, and depletion of antioxidant molecules brings about severe oxidative damage in the melanoma cells treated with ZnO nanoparticles (ZCPAN nanofibers).

**Figure 4:**
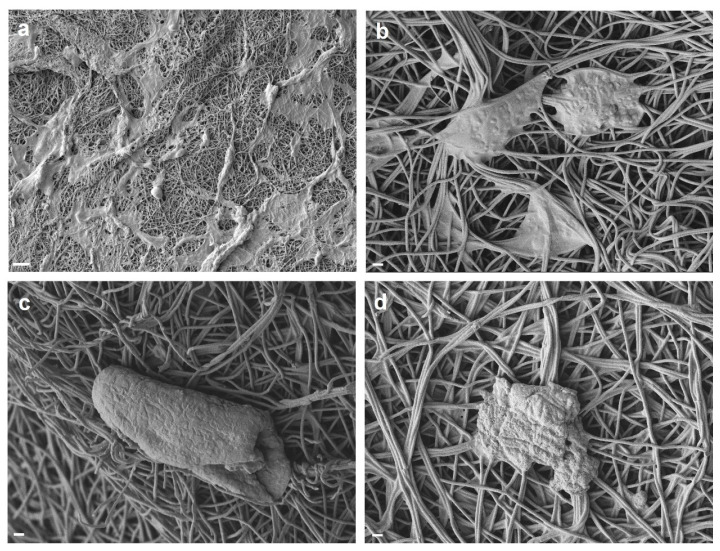
SEM images showing the morphology of the dead A375-M2 melanoma cells on the ZCPAN nanofibers (scale bars represent 10 µm (**a**) and 1 µm (**b**–**d**)). The melanoma cells are of irregular shape, nearly round, and shrunk with deformed plasma membrane, implying their apoptosis.

The ROS induced apoptosis as the main mechanism of melanoma cell death can be also identified through observing the morphology of the dead cells, e.g., via SEM imaging. The surface of apoptotic cells features unique characteristics including blebbing, and their morphology is defined by cell shrinkage and rounding [49]. As seen in Figure 3c, while the live cells feature a spindle-like shape, the morphology of the apoptotic cells changes to round. This observation was further confirmed by SEM imaging in a more precise manner. Figure 4a–d shows the morphology of dead (apoptotic) melanoma cells atop the ZCPAN nanofibrous mats. The cells are obviously shrunk and rounded with plasma membrane deformation. Additionally, as particularly seen in Figure 4b, blebbing of the dead cell membrane, as an indication of apoptosis, is evident. Such an apoptotic feature, aka zeiosis, appears in many cell types [49,50]. A similar morphology for the apoptotic HeLa carcinoma cells was reported by Rello et al. [49]. According to them, early apoptotic cells appear isolated with no plasma membrane connection with their adjacent cells. This feature is also seen in Figure 4c,d, in which shrunk, wrinkled, and isolated dead cells are found across the ZCPAN nanofibrous mats.

## 4. Conclusions

While surgical removal of skin cancer (melanoma) is known as the benchmark in treatment of this disease, the leftover cancer cells might still enter the blood flow, thereby causing distant metastasis or regrowth of a postoperative residual tumor. The most efficient preventive strategy is the development of antimelanoma wound dressings. In the current study, we validated that ZCPAN (ZnO/CAR-hPAN) nanofibers show a strong antimelanoma effect and thus can be used as a proper wound dressing material to prevent recurrence of skin tumor growth. The ZnO nanoparticles in the ZCPAN nanofibers can generate ROS upon UV irradiation and/or release Zn^2+^ ions which can activate ROS production by mitochondria, thereby killing melanoma cells through oxidative stress. On the other hand, the co-existence of CAR and the biomimetic nanofibrous structure of the hPAN nanofiber mat have previously been shown to support wound healing, while inhibiting bacteria activity. Therefore, synergistically, the ZCPAN (ZnO/CAR-hPAN) nanofibrous wound dressing can not only promote skin regeneration (thanks to CAR and hPAN nanofibrous structure) but also inhibit tumor growth (thanks to ZnO nanoparticles). This biomimetic multifunctional nanofibrous wound dressing can provide an effective, post-surgical treatment for melanoma.

## Data Availability

The data that support the findings of this study are available from the corresponding author upon reasonable request.
